# Prevention Interventions of Alcohol Problems in the Workplace: A Review and Guiding Framework

**Published:** 2011

**Authors:** Genevieve M. Ames, Joel B. Bennett

**Keywords:** Alcohol abuse, work-related alcohol and other drug issues, prevention, workplace environment, workplace-based prevention, workplace interventions, employee assistance programs, health promotion, social health promotion, brief intervention, Web-based intervention, environmental-level prevention, employee, employer

## Abstract

The workplace offers advantages as a setting for interventions that result in primary prevention of alcohol abuse. Such programs have the potential to reach broad audiences and populations that would otherwise not receive prevention programs and, thereby, benefit both the employee and employer. Researchers have implemented and evaluated a variety of workplace alcohol problem prevention efforts in recent years, including programs focused on health promotion, social health promotion, brief interventions, and changing the work environment. Although some studies reported significant reductions in alcohol use outcomes, additional research with a stronger and integrated methodological approach is needed. The field of workplace alcohol prevention also might benefit from a guiding framework, such as the one proposed in this article.

Workplace programs designed to prevent and reduce alcohol problems can potentially benefit the employee, the employer, and society in general. In 2007, 8.8 percent of full-time workers overall reported heavy alcohol use (i.e., they consumed five or more drinks on the same occasion on each of 5 or more days in the past 30 days), and 30.2 percent reported binge drinking (i.e., consuming five or more drinks on the same occasion on at least 1 day in the past 30 days) (Substance Abuse and Mental Health Services Administration 2009). As demonstrated in figure 1, when broken down by occupational types, heavy-drinking rates can be much higher in some industries.

Substance abuse is associated with multiple negative workplace outcomes, including absences from work, accidents, turnover, arguments and fighting at work, sleeping on the job, and other sources of productivity loss (Ames et al. 1997; Blum et al. 1993; Dawson 1994; Frone 2006; Lehman and Simpson, 1992; Mangione et al. 1999; Newcomb 1995). Alcohol abuse significantly affects worker productivity. A recent national survey (Frone 2006), using a probability sample of 2,805 employed adults, indicated that work-related impairment directly affects an estimated 15 percent of the U.S. workforce (19.2 million workers). Based on the results of this survey, Frone (2006) estimated that 1.83 percent (2.3 million) of workers drink before work, 7.06 percent (8.9 million) of workers drink during the workday, 1.68 percent (2.1 million) of workers work while under the influence of alcohol, and 9.23 percent (11.6 million) of workers work with a hangover. These estimates are much greater in some occupations versus others.

The estimated costs of alcohol abuse for 1998 (the last year for which costs were estimated for the United States) were $184.6 billion, more than 70 percent of which was attributed to lost productivity ($134.2 billion), including losses of $87.6 billion from alcohol-related illness (Harwood 2000). The comprehensive estimate in 1998 represented a 25 percent increase (3.8 percent per year on average) from the $148 billion estimate for 1992 (Harwood 2000). Using the 3.8 percent per year average increase, the 2010 estimate of overall costs of alcohol abuse are almost double. Employers sustain many of these costs through greater employee turnover (Hoffman and Larison 1999) and reductions in both quantity and quality of work (Mangione et al. 1999). Also, alcohol-related health care costs were estimated at $26.3 billion in 1998 (Harwood 2000). Blose and Holder (1991) found that problem drinkers required injury-related medical treatment 1.6 times more often than people who did not have drinking problems and incurred medical care costs that were three times as high.

The workplace offers many advantages as a setting for preventing alcohol problems. For example, full-time employees spend a significant proportion of their time at work, increasing the possibility of exposure to preventive messages or programs offered through the workplace. Workplace interventions can access specific groups that would otherwise be difficult to reach and, because most people are employed, reach large populations. Employers have a vested interest in keeping their employees healthy and productive. They can therefore use their influence to encourage employees to participate in prevention programs. Many employers offer employee assistance programs (EAPs) to help employees deal with personal problems, including substance abuse, that might adversely affect their work performance, health, and well-being. EAPs generally include assessment, short-term counseling, and referral services for employees and their household members. Although EAPs primarily are treatment oriented, a survey of employee assistance professionals found that most believed that prevention should have a larger role in such programs (Bennett and Attridge 2008). Also, employer health plans may offer confidential alcohol-screening services (Slavit et al. 2009).

It should be noted that small businesses (less than100 workers) tend to be the least likely to have an EAP or health plan (Society for Human Resource Management 2009), to lack health promotion (McMahan et al. 2004), and to also have higher levels of alcohol problem risk (Larson et al. 2007). The 2004 National Survey of Worksite Health Promotion (Linnan et al. 2008) showed that although 90 percent of businesses prohibit alcohol use, significantly fewer (36 percent) offer alcohol screening and support, with small businesses (50–99 employees) significantly less likely (29 percent) than large (more than 750 employees) businesses (71 percent).

This article will present findings from several recent workplace prevention–intervention studies and will focus on those intervention efforts that target all employees, regardless of level of alcohol consumption and problems (i.e., primary prevention strategies).

## Workplace Interventions

Well-developed programs for primary prevention of alcohol abuse in the workplace are more the exception than the rule. However, recent reports suggest that some promising approaches are being developed and implemented. The approaches reviewed here include strategies based in health promotion, social health promotion, and brief intervention, including Web-based feedback interventions, all of which focus on changing individual behavior, as well as environmental interventions, which seek to reduce risk factors by changing the work environment. As the state of the science for workplace prevention is relatively young, the authors present a model that gives equal weight to different approaches until comparatives studies examine their relative effectiveness. This broad suggestion is given to counter tendencies to favor any one approach. For example, health insurance plans currently overly focus on brief interventions for screening and counseling (e.g., Naimi et al. 2006).

### Health Promotion

Through lifestyle campaigns, employers can encourage workers to reduce stress, improve nutrition and exercise, and reduce risky behaviors, such as drinking, smoking, and other drug use. For example, in a study among insurance company workers, Cook and colleagues (2003) tested a program that incorporated substance abuse prevention into both a stress-management program and a nutrition/weight–management program. Participants were randomly assigned to receive either the health program alone or with substance abuse prevention. Both programs were delivered in three group sessions of approximately 45 minutes each.

All participants reported positive changes on measures of stress and healthy eating after the program. All the changes in the stress measures and some of the changes in healthy eating were maintained 8 months later. Importantly, participants in both groups showed similar, significant improvement regardless of the presence of the substance abuse prevention program. For example, the stress-management participants showed significant improvement on the three attitude/perception measures of substance abuse and significant decreases in alcohol and other drug use, regardless of whether they received the substance abuse program. The control group did not show these improvements. The study’s findings suggest that workers can change important attitudes, perceptions, and practices regarding substance abuse if they are exposed to stress-management sessions, regardless of whether explicit substance abuse prevention materials are presented.

Cook and colleagues (2004) evaluated a health-promotion program with substance abuse prevention among 374 construction workers from five sites. Workers were randomly assigned to receive the “Power Tools” program either with substance abuse prevention or without the prevention messages. The program, which used video and print materials and was delivered in seven 45-minute sessions, focused on the adoption of healthful behaviors. Participants in the intervention group showed improvement on stages-of-change measures of smoking and drinking but not on alcohol and other drug use.

Richmond and colleagues (2000) evaluated a workplace lifestyle intervention called Workscreen among 1,206 postal workers in Sydney, Australia. The program included health awareness and brief interventions for high-risk behaviors. In the intervention group, 61 percent of employees overall and 58 percent of those identified as excessive drinkers attended health assessments. Although overall analysis did not reveal reductions in alcohol consumption, women in the intervention group significantly reduced their number of drinks consumed at the 10-month follow-up.

In another study (Heirich and Sieck 2000), researchers assigned 2,000 industrial workers selected through cardiovascular health screening to receive either individual counseling or health education classes. Because of changes in the workplace during the study period, researchers had to create a third intervention group, comprising workers who volunteered for counseling. After 3 years of intervention, 38 percent of drinkers in the highest risk level who received counseling lowered their risk level, compared with 22 percent of drinkers with the same initial risk level who were not counseled. The authors concluded that the counseling intervention, with follow-up, had more impact on behavior change than health education classes.

Lapham and colleagues (2003) evaluated a substance misuse prevention program among 957 health care workers that included health risk appraisal, education, and brief counseling. Although binge-drinking rates were not affected by the intervention, binge drinkers in the intervention group were 2.6 times more likely to report a desire to reduce alcohol use, compared with the preintervention time period and with both time periods in the comparison group.

### Social Health Promotion

Several studies by Bennett and associates (Bennett and Lehman 2001; Bennett et al. 2004) evaluated a classroom-based intervention designed to promote social support and worker peer referral (Bennett et al. 2000). The Team Awareness training focused on enhancing work-group strengths and reducing risks, with attention on workplace climate as a factor in employee drinking. Team Awareness embeds messages about alcohol reduction in the context of team building, stress management, and policy learning. In initial studies with two municipalities, Bennett and colleagues (Bennett and Lehman 2001; Bennett et al. 2004) randomly assigned employees to either an 8-hour psychosocial skills–building course, a 4-hour informational training, or no intervention. Team Awareness promoted responding to problems and encouraged alternatives to the social bonding achieved through drinking. The informational training consisted of a review of alcohol policies, EAP assistance, and drug testing. The first study (Bennett and Lehman 2001) found evidence for increased EAP use (including workers seeking help for alcohol problems), in part as a result of increased peer referral and help seeking. At the 6-month follow-up in the second study (Bennett et al. 2004), employees in both intervention groups reported reduced problem drinking, and the rate of change (45 percent) for the Team Awareness group differed significantly from that of the control group, which had no changes in problem drinking. The Team Awareness group also reported significant improvements in the drinking climate. Follow-up analysis suggested that Team Awareness training may be more effective for work groups that have a more temperate than alcohol-tolerant work climate (Reynolds and Lehman 2008).

A fourth study (Patterson et al. 2005) adapted Team Awareness for small business workers in high-risk industries. A randomized control study assessed the impact of both Team Awareness and a health promotion program on worker methods for unwinding from stress after work. Self-reports of using substances (alcohol and drugs) to unwind and healthy unwinding (e.g., call or spend time with friends or exercise) were examined 2 weeks before and after the training. Although there was no effect on using substances to unwind, Team Awareness participants showed increased use of positive unwinding compared with control subjects.

**Figure 1:**
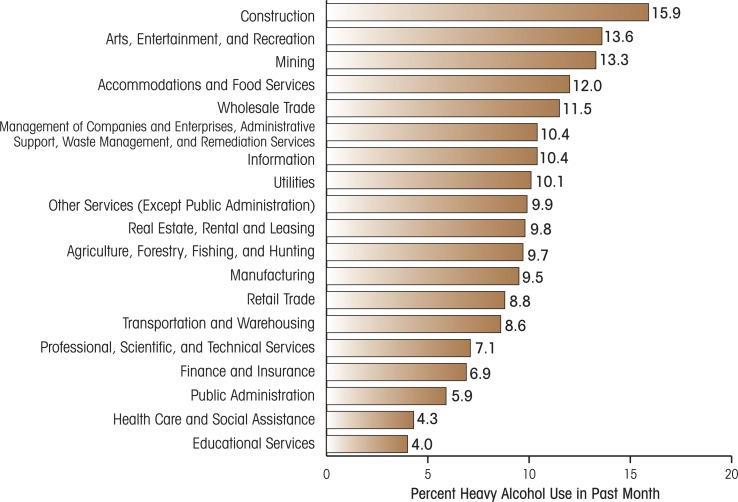
Past-month heavy alcohol use among full-time workers aged 18 to 64, by industry categories: 2002−2004 combined. SOURCE: SAMHSA Analytic Series: A-29. No permission required. Larson, S. L.; Eyerman, J.; Foster, M.S. and Gfroerer, J.C. Worker Substance Use and Workplace Policies and Programs (DHHS Publication No. SMA 07–4273, Analytic Series A–29). Rockville MD: SAMHSA, Office of Applied Studies, 2007.

In a more recent pair of studies (Bennett et al. 2010*a*; Broome and Bennett 2011), Team Awareness was adapted for use with young restaurant workers, a high-risk occupation for heavy drinking (see figure 1). The adapted intervention (Team Resilience), delivered in three 2-hour sessions, included elements to foster social support and consideration, personal confidence, accountability, coping, and stress management. The first study evaluated the program among 124 workers aged 16 to 34 years and found increased awareness of alcohol and other drug risks, help seeking, and personal resilience. The second study used a cluster-randomized trial, with 28 stores from a national casual-dining restaurant chain and 235 employees. Rates of heavy drinking, recurrent heavy (“binge”) drinking, and work-related alcohol incidents (e.g., working while under the influence of alcohol) were assessed at baseline and again at 6 and 12 months. Workers in trained stores reported significantly greater decreases in recurring heavy drinking and work-related problems with alcohol than workers in control stores. In the intervention group, the odds of recurring heavy drinking declined by about one-half, and the number of work-related problem areas declined by one-third after training. Additional analysis revealed that the Team Resilience training also reduced work and personal stress at 6- and 12-months’ follow-up (Petree et al. in press).

### Brief Interventions

Brief interventions typically involve personal assessment of an individual’s drinking rates and related problems as well as feedback about health risks (Bien et al. 1993). These interventions typically have been studied in medical settings and found to be effective there (e.g., Babor et al. 2007) and recently have been applied in work settings (Bray et al. 2009; Hermansson et al. 2010; McPherson et al. 2009; Osilla et al. 2008). Miller and Rollnick (1991) identified six common elements of brief interventions, represented by the acronym FRAMES. These include providing Feedback on personal risks, stressing the importance of taking personal Responsibility for changing one’s behavior, giving Advice to change when appropriate, providing a Menu of options for change, relaying Empathy, and eliciting a sense of Self-efficacy toward making a change successfully.

Brief therapies tend to be physician oriented, whereby an employee, showing signs of alcohol abuse, is more likely to receive screening from their primary care physician (Bertholet et al. 2005; Fleming et al. 2002) than from within the workplace itself (McPherson et al. 2009). Apgar (2003) suggests that brief therapies are most effective when workers have ready access to treatment; support from EAPs or other employee programs; strong family, work, and community ties; substance use problems of short duration; desire to minimize disruption of work and family life; a strong motivation to change; and confidence that their therapy will reduce their substance use. In their review of brief therapies, Slavit and colleagues (2009) claim it as the most cost-effective clinical preventive service and that only 20 percent of employer-sponsored health plans offered such services in 2006 (Bondi et al. 2006; Maciosek et al. 2006).

Brief interventions also can include alcohol education and motivational-enhancement techniques to stimulate behavior change. A few studies have evaluated brief interventions (consisting of one to three sessions) in the work-place. In a study of 155 employees at a food and retail service company, Anderson and Larimer (2002) randomly assigned participants to either a brief alcohol abuse prevention program, featuring personal feedback, alcohol education, and skills training, or a no-treatment control group. Female problem drinkers who received the intervention were more likely than those in the control group to reduce alcohol-related negative consequences at the 6-month follow-up. The results suggested that trained participants also reduced drinking frequency at follow-up.

Walters and Woodall (2003) evaluated a brief intervention conducted by mail among 48 employees at a manufacturing company. Drinkers were either assigned to receive mailed feedback on their drinking immediately or after an 8-week waiting period. Participants were assessed by mail at baseline and after 8 and 16 weeks. After viewing their feedback, participants reported that making a change was more important to them, but they did not have a corresponding increase in confidence that they would succeed in making a change. Participants also reported significant decreases in alcohol consumption after receiving the feedback, and these changes were mediated by participants’ increased perceptions regarding the “riskiness” of alcohol consumption.

Strategic Brief Interventions or Strategic Brief Intervention and Referral to Treatment (SBIRT) (Babor et al. 2007) is a form of brief counseling using standardized screening instruments, following specific guidelines, and follow-up. SBIRT is a promising area for understanding what works, but more research is needed on how this process transfers or translates into and is effective in work settings (Greenwood et al. 2010). McPherson and colleagues (2009) conducted a nonrandom sample survey of employers and vendors regarding their use of strategic brief interventions or SBIRT and found little evidence for such systematic use. Of 265 employers surveyed, 29 percent use any type of alcohol screening, and, of these, 60 percent (18 percent of all surveyed) provide brief interventions. Even fewer (less than 3 percent) used a standard screening tool or followed a systematic procedure that could be described as a strategic brief intervention.

## Web-Based Interventions

Five studies have evaluated the effectiveness of interventions delivered via the Internet to adult workers. Such interventions have the advantage of allowing employees to access the intervention anytime they want and in private to avoid disclosing a potential alcohol problem. Doumas and Hannah (2008) evaluated the efficacy of a Web-based personalized-feedback program delivered in the workplace to 124 young adults (i.e., aged 18 to 24 years). Participants were randomly assigned to either receive Web-based feedback, Web-based feedback plus a 15-minute motivational interviewing session, or to a control group. The Web-based intervention, designed to reduce high-risk drinking by providing normative data regarding an individual’s drinking and the risks associated with drinking, is free to the public and available at www.CheckYourDrinking.net. Participants who received either intervention reported significantly lower levels of drinking than those in the control group at the 30-day follow-up. Participants who were classified as high-risk drinkers (those reporting at least one occasion of binge drinking during the previous 2 weeks at the initial assessment) reported the greatest decreases in drinking between initial assessment and the 30-day follow-up assessment. No differences were found between the two intervention groups, indicating that the addition of a 15-minute motivational interviewing session did not increase the efficacy of the Web-based feedback program.

The U.S. Department of Defense has evaluated a Web-based alcohol intervention called Program for Alcohol Training, Research, and Online Learning (PATROL) among active-duty military personnel. Two Web-based alcohol interventions were adapted for use in the military and tested at eight military installations. Volunteer participants completed a baseline assessment of alcohol use and associated problems and were then assigned to one of four intervention groups: (1) Alcohol Savvy (AS); (2) Drinker’s Check-Up (DCU); (3) the “risk level” condition, where high-risk drinkers were assigned to the DCU and low-risk drinkers were assigned to the AS; or (4) control. Across the installations, 3,912 participants completed the baseline survey and 1,371 completed the 1-month follow-up survey. Results showed that participants who completed one of the programs (i.e., either AS or DCU) had significant reductions on multiple measures of alcohol use compared with control participants. Initial analyses suggested no significant difference in the relative effectiveness of the three program conditions (Pemberton 2007).

Matano and colleagues (2007) studied a Web-based feedback intervention among 145 employees with low or moderate risk for alcohol problems at a company in Silicon Valley, California. All participants were given access to a Web site that provided general information about alcohol use and its effects and feedback on their levels of stress and use of coping strategies. Participants randomly assigned to receive the full feedback intervention also received individualized feedback about their risk for alcohol-related problems. At the 3-month followup, results showed some reductions in drinking among participants who received individualized feedback, although because of the low participation rate (2.7 percent), the sample size was inadequate for determining statistical significance.

Two recent studies also support the use of Web-based programs for reducing alcohol risks for adults. In the first, Billings and colleagues (2008) assessed 309 workers from a technology firm who were randomly assigned to receive a Web-based program on stress and mood management or a waitlist control condition. At the 3-month followup, Web participants showed positive movement on a binge-drinking stage-of-change measure and a trend for experimental participants to report a reduction in drug and alcohol use to manage stress compared with control subjects. The second study (Hester et al. 2009) did not target workers within the workplace setting, per se. However, it is safe to say that this study (Hester et al. 2009) reached adult workers with signs of alcohol dependence. Adults were recruited to the study through advertising and were randomly assigned to either an Internet-based program (www.moderatedrinking.com) and use of the online resources of Moderation Management (MM) (www.moderation.org) or to use of the online resources of MM alone. Results at the 3-month followup indicated that both groups significantly reduced their drinking as well as alcohol-related problems (Drinker’s Inventory of Consequences).

## Interventions Focused on the Work Environment

The environmental approach to research and prevention of workplace drinking problems considers the differences between individual and occupational influences on drinking behavior. Ames (1993) described a cultural model with four interacting conceptual areas for research and prevention of work-related heavy drinking: quality of work life (e.g., stress, alienation, and job satisfaction), social control (policies, visibility, and mobility), alcohol availability (physical and social), and the later addition of social/cultural norms (alcohol beliefs, traditions, and rituals) (Ames et al. 2000). The general hypothesis of this approach is that elements of a work culture and environment, which may vary in context, have the potential to put individuals at risk for problematic drinking and therefore put the workplace at risk for costly work-related problems. Once identified and understood, workplace characteristics that encourage or permit the development and maintenance of undesirable drinking behaviors can be changed to reduce rates of problem drinking in the whole population (see the sidebar on pp. 180) for an example of how this approach has been applied to reduce drinking among young U.S. Air Force personnel). Studies have lent support to the availability (Ames and Grube 1999) and social-control (Ames et al. 2000) components of the model in showing that lowered social and physical accessibility to alcohol and stricter and unambiguous alcohol policies reduce undesirable drinking practices that occur just before coming to work, on the job, and during breaks. A natural experiment testing the efficacy of all components of the work environment model, emphasizing characteristics of social control and alcohol availability, came from a study that compared 12,000 employees in two different manufacturing plants in the same Fortune 500 industry and union but with different management cultures and different approaches to alcohol and drug policy. One approach exemplified a traditional U.S. management–union organizational culture, and the other exemplified an innovative Japanese management–U.S. union culture. Quantitative and qualitative findings described significant differences in drinking at work between the two plants. The U.S.-managed plant, as a result of an adversarial labor relations climate, had an ambiguous and weakened policy embedded in complex organizational barriers to policy enforcement. The foreign-managed plant had an unambiguous policy with few barriers to enforcement. In addition, social and physical availability of alcohol was high in the traditionally managed plant and almost nonexistent in the comparison plant. The foreign-management plant successfully initiated changes in the organizational structure and work culture that limited access to alcohol at work, removed barriers to strict alcohol-related policies and enforcement, and allowed for alcohol and drug testing with cause. The rates of work-related drinking (i.e., drinking before coming to work, during breaks, and on the job) were dramatically different (28 percent for the first plant and 3 percent for the second). The qualitative explanations of differences in these two work cultures highlighted five primary issues or strategies that held potential for environment–focused intervention in other occupational settings (see Ames et al. 2000).

However, it should be noted that strengthening policy language and enforcement is complex, and reluctance to make changes in policy is more a factor of the organizational structure (e.g., management and/or union) than employee resistance. Using the Ames model as a guide, another study that surveyed 7,255 supervisors across seven corporations provided evidence that managers perceive personal, interpersonal, and organizational barriers to enforcing alcohol policy. The lower the manager is in the hierarchy, the more likely he/she is to perceive barriers; furthermore, women managers and first-line supervisors encountered the most barriers (Bell et al. 1996). In the context of that same study, a survey of 6,370 employees at 16 corporate work sites showed that 65 percent of respondents supported pre-employment drug testing, 81 percent supported policies that allowed for testing after an accident, and 49 percent supported random testing. Support was consistent across hierarchy (managers, supervisors, and workers), and support for worksite alcohol testing was highest among blue-collar workers whose jobs involved manufacturing or exposure to worksite hazards (Howland et al. 1996).

## Summary and Recommendations for a Guiding Framework

On the basis of this review of relatively recent research, it seems that approaches aimed at preventing alcohol problems and evaluations of interventions in the workplace have met with some, if not limited, success, and still are in developmental stages. Several studies that integrated alcohol interventions into health promotion programs using a combination of educational, counseling, and brief intervention strategies reported marginally successful results. Drinking rates overall were not significantly reduced; one of these studies reported reduced number of drinks by women only, and another a stated desire by some binge drinkers to reduce alcohol use but saw no actual reduction. The studies that fell into the social health promotion category evaluated skill-building intervention strategies. One successful approach was Bennett and colleagues’ “Team Awareness” program (Bennett and Lehman 2001), which has shown effectiveness with municipal workers, small businesses, and restaurants and also has been recently adapted for electricians and the Youth Corp (Bray et al. 2011). The study of small businesses is important given the lack of programs for this very large segment of the economy. Studies showed reductions in problem drinking, job-related drinking, and work-group drinking climates; increased awareness of risks, help seeking, and resilience; and increased EAP use. The brief intervention approach, variously emphasizing educational, skill building, health risks, self-efficacy, and personal feedback strategies, has been used effectively in medical settings, and early results in the workplace are mixed: women problem drinkers were more likely than control-group women to reduce negative consequences, and participants in the face-to-face, mail, and Web-based interventions were more likely to reduce drinking frequency than those in the control group, but the results differed among the various strategies used for each intervention. Other Web-based approaches that embed alcohol-reduction messages as part of a general health promotion (and stress management) also show promise.

Evaluation of the environmental approach with a natural intervention between two large plants in a Fortune 500 industry used strategies that incorporate changing of identified risk factors embedded in the work culture rather than strategies that are targeted exclusively toward changing individual behavior. The results showed significant differences (28 percent compared with 3 percent) in work-related drinking rates in the plant that made changes in the work culture from the one that did not make changes, and with that outcome, it could be considered a highly successful approach.

All of the strategies that focused on changing individual risk factors and, thereafter, individual drinking behavior, showed some measure of success. As demonstrated in this review, the educational technique, when combined with the brief intervention program, has perhaps the most potential. In terms of improvement, the educational approach, by far the most prevalent program in most work organizations, might benefit from comparison with all of the current intervention approaches, instruments, and results and from there move on to a united effort for an improved design. However, the downfall to these approaches will occur eventually if the programs are not sustained over time and if obstacles (e.g., lack of organizational support and cooperation in implementing follow-up interventions) are not overcome.

Although the approach that focuses on changing the work environment as a means of changing work-related drinking behavior was clearly successful, the limitation here is that only work-related drinking was affected, rather than overall drinking rates, which were similar between the two plants. For purposes of primary prevention, this approach might attain more expansive results if it was blended with one or more of the programs that focus on individual attributes, selecting from among health promotion, social health promotion, and brief interventions. Furthermore, as strongly recommended by Roman and Blum (2002), the involvement of EAPs in primary prevention activities could prove to be a productive strategy.

Importantly, evaluations of the various strategies reviewed here—health promotion, social health promotion, brief interventions, Web-based interventions, and environment strategies—have proceeded in a piecemeal basis without consideration that each strategy varies in its potential reach and overlapping levels of intervention. Such consideration is important because programs that target individual-level behavioral change (e.g., SBIRT) may occur in the context of group processes (e.g., Team Awareness) or wider environmental strategies (e.g., policy enforcement). Likewise, factors like Internet access, health insurance, and EAP programming vary greatly from one organization to another. In short, there is lack of a contextual framework that could guide studies comparing the effects of different interventions.

**Figure 2:**
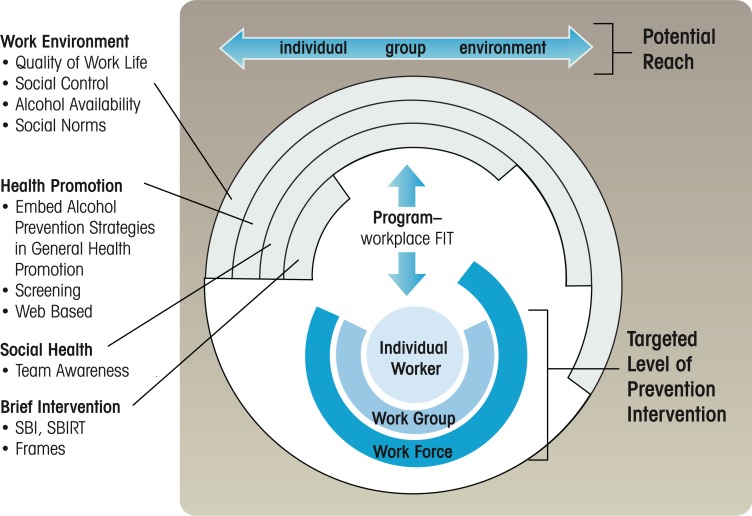
Hypothetical framework for comparing and integrating strategies.

A framework is recommended for guiding thought about future prevention efforts, potentially coordinating evaluation studies, integrating approaches, and ultimately determining which types of programs are most effective (see figure 2). The workplace is a complex setting subject to different factors that may influence program utilization and effectiveness. The framework includes three perspectives to help organize these factors: the target of the intervention; program reach (and overlap); and program fit. First, researchers could benefit from distinguishing the targeted level of the prevention intervention: the individual; the work group; and/or the work force as a whole within the organization. Brief interventions, provided on a one-on-one or Web-based format, tend to be associated with individual change. In contrast, environmental strategies (e.g., alcohol-control policies, monitoring social and physical availability of alcohol during work hours, changes that reduce stress) target the workforce as a whole and changing behavior in the population. However, these tendencies are not necessarily always true because there may be overlapping effects. Policies stemming from environmental strategies may expand or even mandate programming for individual health promotion or screenings, and social health programs may reduce barriers to EAP use where screenings and alcohol treatment programs occur.

Second, programs vary in their potential reach such that they overlap in different ways for different workplaces. Environmental programs have the potential to reach the widest group of individuals (the workforce) with the least or greatest effort, depending on the level of cooperation from management and/or unions. However, brief interventions, in addition to prevention, may reach more individuals who have serious alcohol problems on an in-depth or one-to-one basis. However, brief interventions do not happen in a vacuum and may be facilitated by a social health or health promotion approach, just as these approaches may be most effective in conjunction with an environmental strategy. Although it would require a major effort, a multisite or cross-site study with a cluster-randomized methodology (Murray 1998) would help to tease apart these overlapping effects. Worksites would be randomized to receive environment strategies, and groups and/or individuals nested within these settings would also be randomly assigned to receive different types of strategies (e.g., screening with and without social health). Such effort is important as the true effects from isolated research trials may be under- or overestimated when the variance contributed by other factors (e.g., cohesiveness of work group, strength of EAP) is not included. The current research primarily examines the isolated effects of any one of the four strategies described in this article.

The authors provide a framework (see table) to begin thinking about and generating research designs that would go beyond isolated studies to examine unique, combined, and thereafter holistic effects of interventions. On the other hand, the table can be used to help researchers control for or co-vary other factors. Because of the complexities noted, the four strategies may be better thought of as separate features that can be manipulated (or controlled for) in any design rather than alternative intervention types.

Third, figure 2 also posits the concept of “program–organization fit” or the degree to which any one strategy (or combination of strategies) is more likely to fit in the culture, occupation, or industry receiving the intervention. As noted above (see figure 1), occupations vary in their level of alcohol risk and cultural factors (e.g., alcohol availability). Therefore, when studies find better program use and/or effectiveness in one occupation versus another, lack of effects may be attributed to the level of fit rather than an intrinsic feature of the program. As an example, similar to the case with the work environment approach, the Team Awareness program has built-in customization and adaptation so that “fit” is determined prior to the intervention, requiring coordination between researchers and the organization (Bennett et al. 2010*a*). These adaptation efforts may account for its use in diverse settings (e.g., municipalities, small businesses, restaurant workers, the military, electricians, Youth Corp) (Bennett et al. 2010*b*). Likewise, Web-based programs may be a good “fit” in technical or office professions (where workers have Web access; see Matano et al. 2007) and/or where the participating organization can devote fewer resources to the research study than would be needed for an adaptation effort.

In light of the above, research from the workplace stress literature suggests that “systemic” prevention interventions—that use employee involvement and also integrate individual with organizational change—are more effective than either strategy alone (LaMontagne et al. 2007). Efforts to develop programs that seek to change either the individual or the work environment, or both, have more potential when carried out with input from and ongoing, interactive cooperation of management, union leaders, and other key personnel (e.g. EAP, medical, health promotion) and with input from members of the overall employee population. The identification of specific and modifiable intervention strategies that may emerge in the process of working with various entities within the targeted population is crucial for the development of sustainable prevention of alcohol and other drug problems in the workplace. Another important factor in increasing the potential effects of workplace interventions is the need to draw upon findings from empirical studies that have identified environmental risk and protective factors within specific occupational cultures and integrate these findings into strategies for intervention. Knowledge and consideration of cultural phenomenona relevant to drinking behavior in one workplace over another may be critical to both the successful implementation of and better outcomes of an intervention program.

Finally, the guiding framework may be applied to the study of prevention in high-risk populations. This includes the military (see sidebar), especially for those deployed or returning from deployment, as well as young adult and mature workers about to retire. For example, a recent multisite study of young workers (Bray et al. 2011) examined various types of prevention in a restaurant chain, a transportation company, in workforce development, electrician apprentices in a union setting, a large supermarket chain, and a hospital. Process findings suggest that young adults favor prevention messaging that includes multimedia elements (e.g., Internet and video), integration of substance abuse with health concerns, skills training, and tangible outcomes. Other research suggests that preretirees also can benefit from prevention programs (e.g., Bacharach et al. 2008). Researchers who plan to evaluate prevention in these high-risk populations may benefit from considering the elements of the guiding framework: type of strategy; program–workplace fit; and targeted level of the intervention. In this way, they also can systematically build a knowledge base of evidence-based programs for those who need them most.

## Figures and Tables

**Table:** Framework for Generating Research Designs to Assess Effects of Four Types of Strategies

	**Isolated Effects**	**Combined Effects**	**Unique Effects**	**Holistic Effects**
**Work Environment (WE)**	WE vs. Control Group	(WE + other) vs. Control	(WE+HP+SH+B) vs. (HP+SH+B1)	Comparing organizations with different combinations of interventions vs. control
**Health Promotion (HP)**	HP vs. Control Group	(HP + other) vs. Control	(WE+HP+SH+BI) vs. (WE+SH+BI)	
**Social Health (SH)**	SH vs. Control Group	(SH + other) vs. Control	(WE+HP+SH+BI) vs. (WE+SH+BI)	
**Brief Intervention (BI)**	BI vs. Control Group	(BI + other) vs. Control	(WE+HP+SH+BI) vs. (WE+HP+SH)	e.g., (WE+HP) vs (SH = BI) vs. Control

NOTE: This framework is provided as a guide for developing and coordinating specific strategies rather than a recommendation for a full factorial design. Researchers are encouraged to consider how any one type of intervention occurs in the context of other strategic elements.
