# Supplementary material for: Prevention Interventions of Alcohol Problems in the Workplace: A Review and Guiding Framework
Source: Alcohol Res Health. 2011;34(2):175–87. (PMC3860571)
Supplement: Related texts

# Sub article: Prevention in the Military: Early Results of an Environmental Strategy

**Published:** 2011

**Authors:** Genevieve M. Ames, Christopher Spera

This brief sidebar reviews efforts to implement prevention programs within the military. As a workplace, members serving in the United States military at all ranks and all ages have a significant level of responsibility, especially during times of heavy deployments. Today’s high-risk, lengthy, and frequent deployments can pose significant challenges to military members and their families (Hosek et al. 2006) and can lead to increased rates of problem behaviors, including heavy alcohol use (Ames et al. 2007). The risk for problem drinking increases for service members with more frequent deployments and a greater total cumulative length of time deployed (Spera et al. 2011). Ames and her group confirmed positive relationships between environmental and cultural elements of foreign deployment liberties (e.g., norms, traditions and rituals around heavy drinking and raucous behavior, availability of inexpensive alcohol, youth role-modeling older sailors, low to no supervisory control) and drinking rates and problem behaviors for both young adult (Ames et al. 2009) and careerist members of the U.S. Navy (Ames et al. 2007). Since 1980, smoking and illicit drug use has decreased among military personnel but heavy alcohol use has not (Bray and Hourani 2007). According to the 2008 Department of Defense (DOD) Health-Related Behavior Survey, 20 percent of all service members reported heavy alcohol use (i.e., five or more drinks on the same occasion at least once a week in the past 30 days) (Bray et al. 2009), meaning heavy episodic drinking still is common in the military (Stahre et al. 2009). In a recent survey, military personnel aged 18 to 25 and 26 to 35 years had significantly higher rates of heavy drinking than did civilians in those age groups: 26 percent vs. 16 percent and 18 vs. 11 percent, respectively (Bray et al. 2009).

Despite the seriousness of military drinking problems, prevention efforts that have undergone rigorous evaluation are minimal. Strategies to prevent alcohol- related consequences, such as driving-under-the- influence (DUI) checks, are used in the military, but are not rigorously evaluated (Moore and Ames 2009). Spera and colleagues (2010) reported on an intervention using environmental strategies to reduce drinking and alcohol-related problems among 18- to 25- year-old active-duty Air Force (AF) members in five communities with local Air Force bases (AFBs). The Office of Juvenile Justice and Delinquency Prevention (OJJDP) awarded discretionary grants to five communities in four States as part of the Enforcing Underage Drinking Laws initiative to fund these environmental strategies. The intervention targeted active-duty members living both on and off base. A broad-based coalition (e.g., law enforcement officials, government officials, alcohol and beverage commission representatives, and AF human-service providers) was involved in implementing the intervention across a 3-year period. The five communities were Phoenix, Arizona/Luke AFB; Tucson, Arizona/Davis-Monthan AFB; Honolulu, Hawaii/Hickam AFB; the greater Sacramento area in California/Beale AFB; and Great Falls, Montana/Malmstrom AFB.

Intervention activities included the following:

- Enforcement aimed at reducing the social availability of alcohol, including at least three operations per year to verify drinkers’ identifications and at least two controlled party-dispersal operations per year;
- A minimum of two to three compliance checks (using covert underage buyers) per year at alcohol retailers near the base and in areas frequented by underage active-duty members to ensure that the establishments were not selling alcohol to underage active-duty members;
- Increased number and frequency of DUI checks in the community, including a minimum of at least two DUI patrol operations per year targeted at youth alcohol parties and subsequent driving in and around their respective areas and communities;
- Working to educate State lawmakers about changes to policies and laws that can affect underage drinking (e.g., orienting the photos on licenses of underage and adult drivers differently, such as head-on vs. profile or left side vs. right side);
- Development and deployment of a community- based awareness and media campaign to reduce drinking, including heavy drinking; and
- Offering alternative activities that do not include drinking (e.g., sports activities).

The intervention activities were implemented at a frequency proportional to the size of the community (e.g., Arizona, the most urban site, conducted shoulder- tap operations in as many as 80 locations per year). Each community also received training and technical assistance from the Underage Drinking Enforcement Training Center at the Pacific Institute for Research and Evaluation. This assistance included information about best practices in reducing alcohol use through environmental approaches, hosting teleconferences, and providing hands-on training and technical assistance for coalition development and intervention implementation efforts.

This intervention was evaluated based on the rate of alcohol problems, as measured by the 10-question Alcohol Use Disorders Identification Test (AUDIT), developed by the World Health Organization (Babor et al. 2001). Individuals who scored eight or higher on the AUDIT were identified as problem drinkers. To determine the effects of the intervention, the researchers used data from the Air Force Community Assessment survey to assess the prevalence of problem drinking among junior enlisted personnel for each site at pretest (2006) and posttest (2008). Each intervention site was compared with a corresponding comparison community of similar mission, size, urban or rural typology, and rate of problem drinking at baseline and with the AF’s overall prevalence of problem drinking.

In the AF overall, the rate of individuals classified as problem drinkers decreased significantly from 20.4 percent at pretest to 13.8 percent at posttest. Broken down by location, the percentage of problem drinkers at the Phoenix, Arizona/Luke AFB community was not significantly different from either the comparison site or the AF overall at pretest, but at posttest, was 7.7 percentage points lower than the comparison site and 5.9 percentage points lower than the AF overall. When comparing the changes across time, the Phoenix, Arizona/Luke AFB community had a decrease of 13.6 percentage points in problem drinkers, compared with a 1.9 percentage point decrease for the comparison site and 6.6 percentage point decrease for the AF overall.

At the Tucson, Arizona/Davis-Monthan AFB community, the percentage of problem drinkers was not significantly different from either the comparison site or the AF overall at pretest and posttest, but comparing the changes across time, showed a decrease of 9.8 percentage points in problem drinkers, compared with a 11.2 percentage point decrease for the comparison site and a 6.6 percentage point decrease for the AF overall. When comparing these changes across time, the prevalence rate change was not significantly different from the change at the comparison site and the AF overall.

For the Honolulu, Hawaii/Hickam AFB community, the percentage of problem drinkers was not significantly different from either the comparison site or the AF overall, but at posttest, the percentage was 7.0 percentage points lower than the comparison site and 4.3 percentage points lower than the AF overall. When comparing the changes across time, there was a decrease of 9.4 percentage points in problem drinkers, compared with a 5.8 percentage point decrease for the comparison site and a 6.6 percentage point decrease for the AF overall. In the Sacramento, California/Beale AFB community, the percentage of problem drinkers was not significantly different from either the comparison site or the AF overall at pretest and posttest, but across time there was a decrease of 8.1 percentage points in problem drinkers, compared with a 9.3 percentage point decrease for the comparison site and a 6.6 percentage point decrease for the AF overall. When comparing these changes across time, the prevalence rate decline was not significantly different from the change at the comparison site and the AF overall.

Finally, in the Great Falls, Montana/Malmstrom AFB community, the percent of problem drinkers was not significantly different from either the comparison site or the AF overall at pretest, but at posttest, the percentage of problem drinkers was 12.8 percentage points less than the comparison site but 5.5 percentage points more than AF overall. Comparing the changes across time showed a decrease of 5.3 percentage points in problem drinkers, compared with an increase of 11.3 percentage points for the comparison site and a decrease of 6.6 percentage points for the AF overall. When comparing these changes across time, the prevalence rate declined at the Malmstrom AFB to a larger degree than at the comparison community.

## Conclusions

This sidebar presents the early findings of an intervention evaluation. The authors point out that the rate of problem drinking in the AF overall for junior enlisted personnel decreased significantly (6.6 percentage points) over the evaluation period, which could be attributed to AF-wide prevention activities such as the Culture of Responsible Choices program, among other factors. Although all five sites showed decreases in the percentage of junior enlisted personnel reporting problem drinking over the evaluation period, the most promising early findings were from the two Arizona communities (i.e., a 13.6 percentage point decrease at the Phoenix/Luke AFB community and a 9.8 percentage point decrease at the Tucson/Davis-Monthan AFB community). The authors note that these changes represent promising, but not necessarily causal, relationships between the intervention and survey results. They suggest that Arizona was particularly successful for a number of reasons, including the speed with which the coalition was developed and the intervention activities that were implemented. The two Arizona communities also used guidance from the Underage Drinking Enforcement Training Center at the Pacific Institute for Research and Evaluation, input from Federal partners (i.e., the OJJDP and the National Institute on Alcohol Abuse and Alcoholism [NIAAA], and U.S. AF), and best- practice guidelines from the Substance Abuse and Mental Health Services Administration on building State-based coalitions to promote community prevention. Related to this, Arizona had an underage-drinking task force in place before the intervention began. In addition, these two communities hired an outside local partner (i.e., Pima Prevention Partnership) to help implement their activities. Given that both of these communities are in urban areas, they were able to more readily identify and offer alternative activities to drinking for young active- duty members, compared with sites in more rural areas, where the number of alternative activities may be perhaps limited. Finally, the two Arizona communities received strong support from base-level leadership.

Additional research will continue to evaluate the rates of problem drinking in these communities over time to determine whether the changes reviewed here persist.

Another prevention project—a collaboration between prevention researchers and the U.S. Navy—resulted from three separate studies (funded by the NIAAA and the DOD) on environmental risk factors for problem drinking, drug and tobacco use, and health and social problems related to drinking (Ames et al. 2007; 2009; Cunradi et al. 2005, Moore et al. 2007). By invitation from U.S. Navy commands at Norfolk, VA and the Great Lakes Training Center, Illinois, Ames and colleagues presented to a selected group of military and civilian personnel their findings on rates of substance use and related problems, work-related risk factors, and specific guidelines for prevention programming. The research projects and guidelines for prevention were organized around a conceptual model that includes environmental factors of alcohol availability, policies, work stress, and cultural norms (Ames 1993; Ames et al., 2007; 2009). Electronic and hard copies of research results and suggested guidelines for prevention were provided to the Inspector General of the U.S. Navy and other relevant personnel. Although the Inspector General stated in writing that these findings and guidelines would be incorporated into system-wide substance abuse prevention efforts, to our knowledge, the outcomes have not been evaluated.

## References

[b61-arh-34-2-175] Ames G, Cunradi C (2005). Alcohol use and preventing alcohol-related problems among young adults in the military. Alcohol Research & Health.

[b62-arh-34-2-175] Ames GM, Cunradi CB, Moore RS, Duke M (2009). The impact of occupational culture on drinking behavior of young adults in the U.S. Navy. Journal of Mixed Methods.

[b63-arh-34-2-175] Ames GM, Cunradi CB, Moore RS, Stern R (2007). Military culture and drinking behavior among U.S. Navy careerists. Journal of Studies on Alcohol and Drugs.

[b64-arh-34-2-175] Bray RM, Hourani LL (2007). Substance use trends among active duty military personnel: Findings from the United States Department of Defense Health Related Behavior Surveys, 1980–2005. Addiction.

[b65-arh-34-2-175] Bray RM, Pemberton MR, Hourani LL (2008). Department of Defense Survey of Health Related Behaviors Among Active Duty Military Personnel.

[b66-arh-34-2-175] Cunradi C, Ames G, Moore R (2005). Prevalence and correlates of interpersonal violence victimization in a junior enlisted Navy cohort. Violence and Victims.

[b67-arh-34-2-175] Hosek J, Kavanagh J, Miller L (2010). How deployments affect service members [article online].

[b68-arh-34-2-175] Moore RS, Ames GM (2009). Young impaired drivers: The nature of the problem and the strategies being used in the military. Young Impaired Drivers: The Nature of the Problem and Possible Solutions. Transportation Research Circular E-C132.

[b69-arh-34-2-175] Moore RS, Ames GM, Cunradi CB (2007). Physical and social availability of alcohol for young enlisted naval personnel in and around home port. Substance Abuse Treatment, Prevention, and Policy.

[b70-arh-34-2-175] Spera C, Barlas F, Thomas R (2011). Relationship of military deployment recency, frequency, duration, and combat exposure to alcohol use in the Air Force. Journal of Studies on Alcohol and Drugs.

[b71-arh-34-2-175] Spera C, Franklin K, Uekawa K (2010). Reducing drinking amongjunior enlistedAir Force members in five communities: Earlyfindings of the EUDL program’s influence on self-reported drinking behaviors. Journal of Studies on Alcohol and Drug.

[b72-arh-34-2-175] Stahre M, Brewer R, Fonseca V, Naimi T (2009). Binge drinking among U.S. active-duty military personnel. American Journal of Preventive Medicine.
